# Satellite measurements reveal strong anisotropy in spatial coherence of climate variations over the Tibet Plateau

**DOI:** 10.1038/srep30304

**Published:** 2016-08-24

**Authors:** Deliang Chen, Yudong Tian, Tandong Yao, Tinghai Ou

**Affiliations:** 1 Regional Climate Group, Department of Earth Sciences, University of Gothenburg, Gothenburg, S-405 30, Sweden; 2 NASA GSFC and ESSIC, University of Maryland, College Park, MD 20742, USA; 3 Institute of Tibetan Plateau Research, Chinese Academy Sciences, Beijing, 100101, China

## Abstract

This study uses high-resolution, long-term satellite observations to evaluate the spatial scales of the climate variations across the Tibet Plateau (TP). Both land surface temperature and precipitation observations of more than 10 years were analysed with a special attention to eight existing ice-core sites in the TP. The temporal correlation for the monthly or annual anomalies between any two points decreases exponentially with their spatial distance, and we used the e-folding decay constant to quantify the spatial scales. We found that the spatial scales are strongly direction-dependent, with distinctive patterns in the west-east and south-north orientations, for example. Meanwhile, in the same directions the scales are largely symmetric backward and forward. Focusing on the west-east and south-north directions, we found the spatial coherence in the first is generally stronger than in the second. The annual surface temperature had typical spatial scales of 302–480 km, while the annual precipitation showed smaller scales of 111–182 km. The majority of the eight ice-core sites exhibit scales much smaller than the typical scales over the TP as a whole. These results provide important observational basis for the selection of appropriate downscaling strategies, deployment of climate-data collection networks, and interpreting paleoclimate reconstructions.

The Tibetan Plateau (TP) is the highest and most extensive highland in the world. It features a distinct natural environment with global impact. Major Asian Rivers originate from this region, which makes the understanding of the complex climate variation crucial in assessing water resources/risks. This is of huge importance to one third of the world’s population, both directly through water resource security and indirectly as a result of geopolitical consequences.

The diverse climatic conditions and extreme topographical relief within the region demand extensive meteorological and hydrological observations. However, the entire region has poor coverage of *in-situ* stations. This makes the understanding of key processes important to regional climate and water resources difficult and speculative^1^. This is especially true for precipitation in relation to topography across the TP^2^.

Thanks to the recent availability of long-term, high-resolution satellite-based surface observations, we now have a unique opportunity to study many fundamental characteristics of the key processes with space-borne data. One of these characteristics is the spatial scales of climate variation over the TP, which is largely unknown. How large are spatial coherence of climate variations? Is the spatial coherence independent of orientation? What is the difference in the spatial coherence between surface temperature and precipitation? Answers to these questions can yield much needed guidance on design of climate simulations and observation networks, selection of downscaling strategies, and conducting impact studies. It would also help to put *in-situ* records (e.g. from ice-cores) into perspective. A few existing adjacent ice-cores in the TP have revealed somewhat different accumulation and isotope records, suggesting to small-scale variability^3^,^4^,^5^.

The problem of accurately representing the spatial variability of climate variables arises frequently in a variety of applications, such as in hydrological studies^6^,^7^. For example, given an accuracy requirement on the sampling error of precipitation, the knowledge of precipitation spatial variability directly dictates the deployment of rain gauges. Such spatial variability is usually quantified as the spatial scale of correlation decay. For instance, in an effort to evaluate a station network’s estimate of the daily precipitation over a watershed in northern Vermont, Hendrick and Comer^8^ characterized the spatial variations in daily precipitation in terms of inter-station correlations. They found that the spatial distribution of the correlation centered at a reference gauge is dependent on inter-station distance, azimuth angel, daily rainfall amount, and season. They derived a spatial correlation function and used it to determine the required rain gauge density and configuration for their desired accuracy.

Similar studies over the TP would have been challenging. Mainly due to the inaccessibility of large parts of the TP, The density of the existing meteorological stations is generally low, and a serious gap exists in the central and western areas^9^,^10^. As a consequence, empirical climatic studies for the TP are often limited to using records from scattered operational meteorological stations in the region. This severely hinders our understanding of the regional climate. These factors all provide motivation for increasing use of satellite data, which furnish much better geographical coverage despite their potential limitations such as the accuracy under cloudy and/or snowy conditions and the relatively short period of observation. Satellite data is playing an increasing role in revealing details of the regional climate over the last decade^11^. As an example, in the Himalayan region where rain gauges are limited or nonexistent, satellite-based rainfall estimates can provide useful information on rainfall occurrence, amount, and distribution^12^. By evaluating four high-resolution satellite precipitation datasets against gauge measurements over different climate zones of the TP during 2004 and 2009, Gao and Liu^14^ concluded that high-resolution satellite precipitation datasets are very attractive for studying the hydrologic processes in mountainous areas where rain gauges are generally sparse.

We focus here on quantification of the spatial climate variability over the TP with long-term, high-resolution satellite data. An example showing representativeness of eight ice-core sites in the region (Fig. 1) is provided to demonstrate the usefulness of the study. These ice-core sites have recorded climate conditions over long time periods^14^,^15^,^16^. Better knowledge of the characteristics of the regional climate variability from the current study will greatly help one understands and interprets these ice-core records.

## Results

### Spatial scales of land surface temperature and precipitation

As a common practice, the spatial scale of a climate variable is defined as the e-folding distance of the correlation decay, modeled realistically as an exponential decay function over spatial distance (for detailed definition see Supplementary Fig. S1). Due to possible directional dependence, we need to look at scales in various directions. Figure 2 shows the spatial scales for monthly time series of land surface temperature and precipitation in the four cardinal directions and their two backward/forward combinations. From the figures it is clear that the northward and southward scales are largely symmetric, which justifies our choice to combine the two directions for the later analysis. The same is true for the westward and eastward directions. Thus, we also provide the spatial scales in these two combined directions in the figure and, hereafter, we focus on results oriented in these two main combined directions.

**Figure 1:**
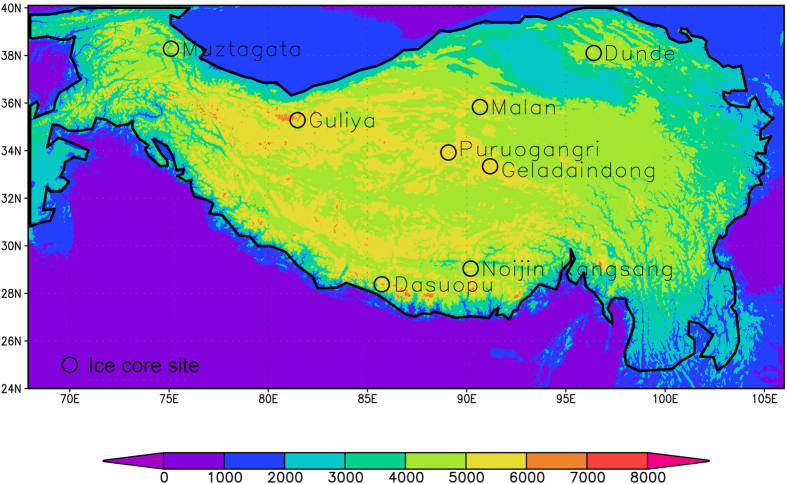
Surface elevation above sea level (Unit: m) for the study area. The solid line indicates the 2000 m contour line designated as the boundary of the Tibetan Plateau (TP). The eight ice-core sites are indicated by the black circles. (Figure was created using Grads version 2.0.2, http://iges.org/grads/).

**Figure 2:**
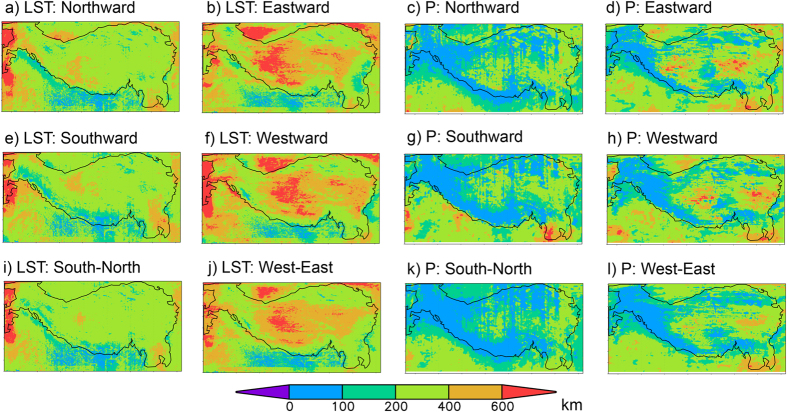
Spatial scales for monthly land surface temperature (LST) (**a,b,e,f,i,j**) and precipitation (P) (**c,d,g,h,k,l**) in four cardinal directions and their two backward/forward combinations: northward, eastward, southward, westward, south-north, and west-east. (Figure was created using Grads version 2.0.2, http://iges.org/grads/).

For land surface temperature, spatial coherence is generally weak along the boundary of the plateau (Fig. 2), especially along the Himalaya mountain range. This is not surprising, as sudden changes in elevation can cause great differences in local temperature and its variation, and high mountains can act as barriers to the transportation of heat and moisture, creating a natural division of different climates and their variations. Generally speaking, the scale in the west-east (W-E) direction is larger than that in the south-north (S-N) direction; this can be explained by the dominant westerly winds and orientation of the valleys of the parallel west-east (W-E) direction mountain ranges such as the Kunlun Mountains, the Tanggula Mountains and the Himalayas Mountains (Fig. 1).

Precipitation over a complex terrain such as the TP can be highly discontinuous in space and time^17^,^18^. Thus a complicated structure and contrasted spatial scale for precipitation in the region is to be expected. The spatial scales for precipitation shown in Fig. 2 confirmed these expectations. As with land surface temperature, there is a similarity for anisotropy in the spatial coherence of the precipitation variations: stronger coherence in the west-east directions and weaker in the south- north directions. Terrain features seem to have a bigger impact on the variation in precipitation than on that of land surface temperature. In addition, there are some common features between the patterns for land surface temperature and precipitation, such as the overall very small spatial scales along the high mountain in the south. This indicates that there are common factors that control both land surface temperature and precipitation. Topography is most likely the leading one.

**Figure 3:**
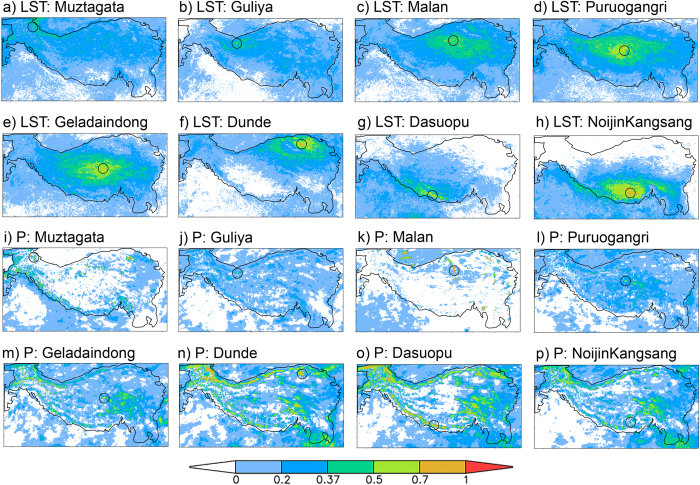
Spatial distributions of the temporal correlation coefficients between each ice-core site and all other grid boxes of the entire study area, for land surface temperature (LST) (**a~h**) and precipitation (P) (**i~p**) respectively. The calculations were done with the monthly data. (Figure was created using Grads version 2.0.2, http://iges.org/grads/).

It has long been established that spatial scale of climate variables varies geographically and depends on the choice of directions^8^; we found that this was, indeed, the case in the current study. The eight selected ice-core sites serve as practical examples. Figure 3 shows spatial distribution of correlation coefficients centered on the eight selected ice-core sites for both annual land surface temperature and annual precipitation time series. Following the definition of the spatial scale illustrated in Supplementary Fig. S1, the correlation coefficients for the ice-core sites contain information about the spatial scale, once the predefined threshold correlation coefficient has been set. These correlation patterns are manifestations of the spatial scales for the corresponding variables in all possible directions. Such knowledge is very useful in identifying key regions influencing the local climate, and for interpreting the spatial representativeness of the ice-core records.

From these maps it becomes clear that the spatial anisotropy is, indeed, an outstanding feature in this region’s climate variability, and such anisotropy varies among the ice-core sites, depending on each site’s location. Again, relative to the precipitation variation, the temperature variation overall exhibits a more symmetrical coherency pattern and has larger spatial coherency. It is also noteworthy that there can be rather high level of small-scale variability in the correlation fields, especially for the precipitation maps. This can be caused by real small-scale climate variability and/or noise in the data used to estimate the correlation. Indeed, as demonstrated by Supplementary Figs S1 and S2, correlation coefficients can fluctuate considerably around the theoretical model assumed for spatial variation. Therefore, the estimated spatial scales in the two main combined directions (Fig. 2) should not be taken as the only spatial coherencies for the study area. Indeed, in some cases there can be fairly big differences between the backward and forward coherences in the same combined directions. The differences in the estimated scales between the two main directions can sometimes also be very large (Table 1).

The possibility of some really small-scale variability is illustrated in Fig. S2 which shows the correlation coefficients of annual precipitation between the Noijin Kangsang grid box and all other grid boxes in the two main directions in the study area. It is clear that the correlation coefficients quickly dropped below the e-folding threshold beyond the first adjacent grid box. However, they increased again after some distances. In that case, the spatial scale was set to be equal to the resolution of the data, although the neighboring areas do show high similarities to the reference grid box (also visible in Fig. 3p). This example demonstrates the importance of small-scale variability due either to reality or to a high level of noise in the data. It may also imply that the theoretical model used in determining the spatial scale is not always valid. The uncertainties associated with the noisy data and/or small-scale variability make us believe that maps like those in Fig. 3 or Fig. 2 are more useful than a spatial scale estimated in a specific direction for revealing the spatial scale around a location. In any case, the sites with an extremely small spatial scale in one direction or more should be further investigated to facilitate better interpretation of their ice-core records. For the sake of simplicity and feasibility, however, in what follows, we will only focus on the spatial scales in the W-E and S-N directions.

**Figure 4:**
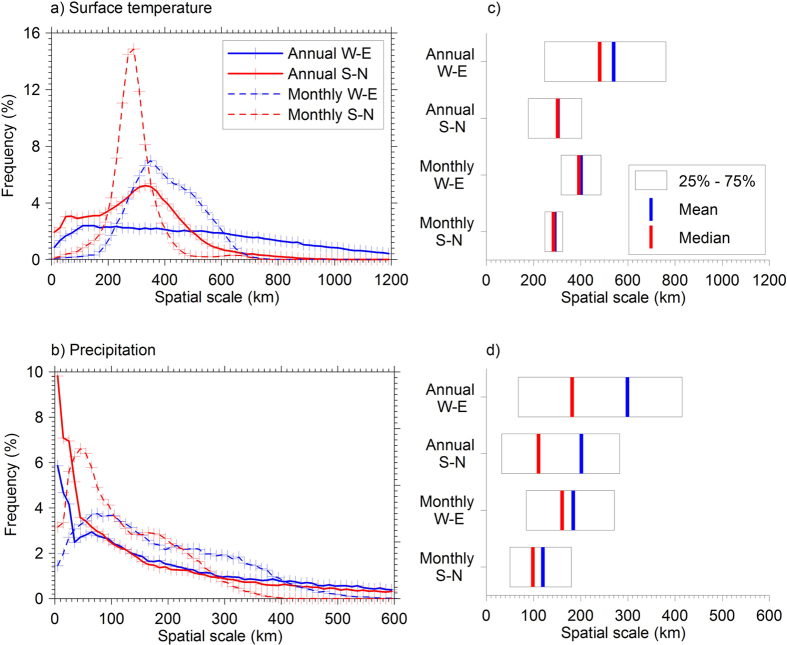
The frequency distributions of the spatial scales for surface temperatures (**a**) and precipitations (**b**) in the W-E and S-N directions for all the grid boxes in the study area. The spatial scale corresponding to the medians of the probability density function is defined as the typical spatial scale. Scales computed from both the annual data (solid curves) and monthly data (dashed curved) are shown. The summary statistics for the spatial scales over the study area for the land surface temperatures and precipitations are also shown in (**c** and **d**) respectively. The boundaries of the box indicate the 25th- and 75th –percentile. The intervals chosen for plotting the histograms are 20 km for the temperature (**a**) and 10 km for precipitation (**b**) respectively.

To summarize the variation in the spatial scales over the TP and to determine a typical spatial scale for land surface temperature and precipitation variations over the TP, we show the frequency distributions of their respective scales across the TP in Fig. 4. Also shown are the associated summary statistics of the estimated scales. In addition to the spatial scales based on the annual means, the spatial scales using monthly means are also shown. For land surface temperature, the distributions roughly follow a normal distribution, with a slightly longer tail on the larger spatial scale side. The medians of the distributions are defined as the typical scales in this study. In Fig. 4 the wide distributions as a general feature remind us of the fairly wide spread of spatial scales depending on where the reference grid box is located. While the spatial scales based on the monthly mean land surface temperature show a distinct peak around the typical scale, the annual data give a relatively flat distribution, indicating more evenly distributed spatial scales over the longer time scale. Figure 4a,c show that the spatial scale of the interannul variation in land surface temperature is generally larger than the month-to-month variability. This is expected, as longer temporal scales tend to be associated with larger spatial scales. Further, the generally larger scale in the W-E direction than the S-N direction is confirmed.

Overall, the typical scale of the annual surface temperature revealed by the satellite data for the entire TP region ranges from 302 to 480 km in the two main directions, and only one out of the eight ice-core sites (Puruogangri) has scales much larger than the typical scale for temperature in W-E direction (Table 1, Fig. 3m).

For precipitation, each frequency distribution of the spatial scale is very different from that of the surface temperature. It to some extent resembles Gamma or lognormal distribution better, with a long tail towards larger scales. There are high percentages of the grid boxes which show the shortest scale (Fig. 4b). Since this feature only appeared in the annual data, it should be taken with caution as indicated by Supplementary Fig. S2. In general, the typical scales are reached within a relatively short distance from the reference grid box compared with those for land surface temperature, indicating smaller scale variations. The typical scale of the annual precipitation variation in the two main directions ranges from 111 to 182 km only (Table 1). Dunde is the only site which exhibits a larger scale than the typical scale in S-N direction.

### Typical spatial scale as a function of a threshold correlation coefficient

The preceding section quantified the characteristics of the spatial scales for the climate variables by using the e-folding correlation as a threshold. In other applications, however, we may need to quantify spatial coherency with a different threshold correlation coefficient^19^. As an example, in paleoclimate reconstruction using tree ring data, one meteorological station closest to the tree(s) used is usually needed to establish and calibrate the reconstruction model^19^. In that case, a higher correlation coefficient than the one used in this study to define the spatial scale is required to find a reasonable proxy for the local climate conditions at the location of the tree(s).

Knowledge about typical scale as a function of any threshold correlation coefficient is also helpful in model-data comparison in the complex terrain^20^. To make the scale analysis presented useful for other applications, we have established the relationship between the threshold correlation coefficient and the typical spatial scale over the TP for the two climate variables with monthly and annual data. This was done by determining typical spatial scales in the two main directions with different thresholds of correlation coefficient ranging from 0.25 to 0.95 at increments of 0.05.

**Figure 5:**
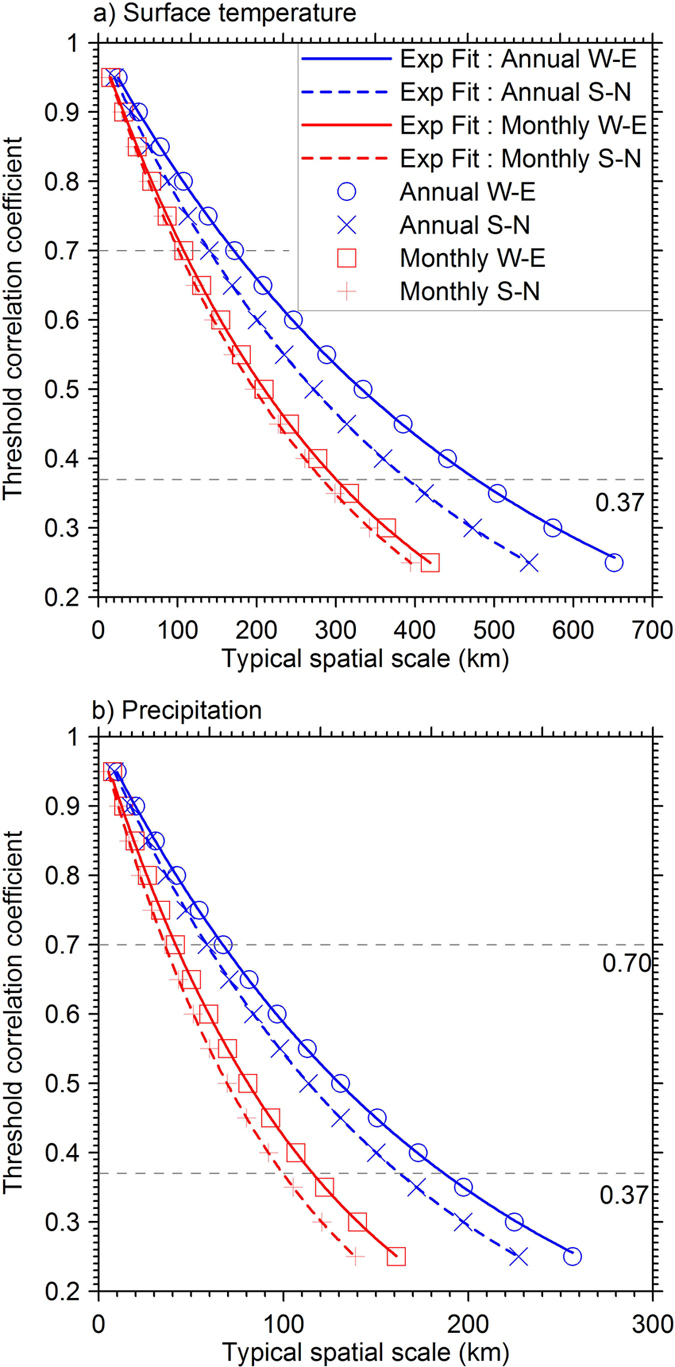
Relationship between the threshold correlation coefficient and the associated typical spatial scale for surface temperatures (**a**), and precipitations (**b**) in the two main directions. The typical spatial scales corresponding to different thresholds of correlation coefficient (from 0.25 to 0.95 with a 0.05 increment) were determined and are represented by the four different symbols. The four lines are fitted exponential functions of the decays of the correlations with increasing distance.

Figure 5 shows the established relationships for both the monthly and annual surface temperature and precipitation. For the TP as a whole (for boundary of the TP see Fig. 1), typical scales decrease with increasing threshold correlation coefficients for both land surface temperature and precipitation. All the relationships can be well illustrated with an exponential function. The typical scales of the two climate variables for the TP are longer along the W-E direction than that along the S-N direction, and spatial scales for the annual data are larger than those for monthly data. All these are consistent with what have been shown in Fig. 4.

## Discussions

We made additional attempts to identify the relationships between the spatial scales of surface temperature and topographic and geophysical features, such as terrain shape, surface roughness, mountain slope and aspect, etc. While we are aware of the complex processes resulting in the specific surface temperature^21^,^22^, it is tempting to examine whether some of these factors can be used to explain the distribution of the spatial scales. However, our investigation did not produce useful results. Some other factors, meteorological, hydrological, and ecological should be considered for future studies.

Another critical issue is related to the fact that the satellite-based precipitation data, CMORPH, tends to have higher uncertainty over our study area than over other areas^23^ due to the complex surface conditions, and for this study we assumed that the errors in the precipitation data are spatially uncorrelated. This can introduce some underestimation of the spatial scales. Although this is a more reasonable assumption than otherwise, the effect cannot be quantified as our understanding of errors in the precipitation data is still not quantitative or complete. The current study could be extended in the future when more reliable precipitation data or error information becomes available.

A question arises about the extent to which the spatial scales determined for the land surface temperature are useful indicators of the scales for the near surface air temperature which is a more convenient climate variable. Since the satellite data only shows the surface temperatures which are different from the air temperature at a height of 2m measured by ground-based meteorological stations, the results based on the satellite skin temperature may not be representative of the surface air temperature. Here we took advantage of the ERA-Interim surface temperature and air temperature at 2m over the study area and explored the relationship between the two at the monthly and annual scales.

Supplementary Fig. S3a shows the spatial distribution of the correlation coefficients between the two monthly time series. To focus on the real co-variation between the two, the seasonal cycles of the time series were removed by subtracting the long term monthly means of the two prior to the correlation. Except for a few cells, there are very high correlations over the entire TP, indicating the usefulness of using the surface temperature to infer spatial scale for surface air temperature across the region. A similar result was also found with the annual data (see Supplementary Fig. S3b). In fact, recent studies^24^,^25^ have confirmed the strong link between the two temperatures. The usefulness of reanalysis surface air temperature for representing the interannual variability of temperature and regional patterns has been demonstrated by Frauenfeld *et al*.^26^ using ERA-40 and station data over the TP. The ERA-Interim air temperature in the study area was also validated with observational data^27^. In another study, Zhong *et al*.^28^ looked at the trend of the LST (AVHRR) and the surface air temperature (ECMWF) over the period from 1982 to 2000 in the TP and found that the two increased at a similar rate (the LST by 0.27 K per decade and the surface air temperature by 0.30 K per decade) over the recent decades.

## Methods

All the data used are from other sources than the authors’ own. Interested readers are welcome to contact the corresponding authors for information about and access to the data.

### Study region and ice-core sites selected

Figure 1 shows the study area. The topography and the locations of the eight ice-core sites selected are also indicated. Supplementary Table S1 gives detailed information about the geographical locations and other details of the ice-core sites. The scale analysis in this study provides hints about the representativeness of the sites with respect to climate variations.

### Reanalysis data

Despite the fact that the existing global reanalysis products do not have enough spatial resolution to quantify the local-scale processes over the study area, they do provide an excellent opportunity to examine the interrelationships among different climatic variables. We used the ERA-interim reanalysis for the period 1979–2014^28^,^29^ to quantify relationships between the surface air temperature and skin temperature, which is comparable to the land surface temperature derived from the satellite datasets. The ERA-Interim reanalysis is an improved version of the ERA-40 reanalysis. An updated ECMWF forecasting model version cycle 31r1 was used with a horizontal resolution of approximately 80 km for ERA-Interim. Aside from the improved resolution over ERA-40, ERA-Interim utilizes four-dimensional variational data assimilation (4DVar) and bias correction of satellite radiance data^29^,^30^, better formulation of background error constraint, new humidity analysis, and improved model physics. ERA-Interim also mostly uses the sets of observations acquired for ERA-40, supplemented by data for later years from ECMWF’s operational archive. ERA-Interim has been proven to be the best among the reanalysis products available for describing temperature and water cycle over the TP^31^,^32^.

### Satellite surface temperature and precipitation datasets

Two satellite-based datasets were used for this study. First, we used the land surface temperature (LST) data, officially designated as the “MODIS/Terra monthly daytime 3 min (0.05-deg) Climate Modeling Grid (CMG) Land-surface Temperature, Collection 5 (MOD11C3.005)”^33^,^34^, available for a period of 14 years (Aug. 2000 through Jul. 2014). LST is a variable that reflects the complex interactions between many climate variables, including air temperature, soil moisture, vegetation cover, etc., as documented, for example, by Jin and Dickinson^21^ using the same LST data.

The second data set is CMORPH 8 km, 30-min precipitation estimates. The name CMORPH refers to the precipitation estimates using the National Centers for Environmental Prediction (NCEP)’s Climate Prediction Center (CPC) MORPHing technique^35^,^36^. This technique uses the high resolution infrared imagery to infer the motion of rainfall patterns between passive microwave scans, and use this advection information to obtain a smooth “morphing” of rain patterns obtained from the more reliable passive microwave based rain retrievals. CMORPH has been fairly extensively validated^13^,^23^,^35^ and its error characteristics are relatively well understood. For example, by comparing four high-resolution satellite precipitation products with gauge measurements over different climate zones of the TP, Gao and Liu^13^ found that CMORPH is close to the ground based observations and ranked top of the four products.

Both data sets were aggregated to monthly total and then the seasonal cycle was removed. We kept their original spatial resolutions (0.05-degree and 8-km, respectively) when we performed the spatial scale calculations.

### Spatial correlation and spatial scale

This study focuses on spatial correlation of temporal variation at monthly or annual scale. For a given grid box and a given climate variable (land surface temperature or precipitation), the monthly/annual mean climate variable was correlated with the same variable for each of the other grid boxes in a given direction for the entire period when observations are available. The correlation reflects the consistency of the temporal variability resolved by the monthly or annual data between the reference grid box and the other grid boxes.

Supplementary Fig. S1 provides an example of a reference taken from one of the eight ice-core sites (Geladaindong). The correlations coefficients between the annual mean temperature for the grid box in which Geladaindong is located and temperature for other grid boxes in the S-N direction were calculated and plotted against the distance from the grid boxes to the ice-core site. As expected, the correlation coefficient generally decreases with increasing distance between the reference grid box and other grid boxes to begin with and then approaches a stable level around which the correlation coefficients fluctuate around zero and the distance no longer plays a significant role. Given a certain level of a threshold correlation coefficient, we can determine the distance within which the significant correlation exists.

The spatial and temporal variability of many climate variables can be modeled, to the first order of approximation, as a first-order Markov process^37^,^38^,^39^. Under this assumption, the correlation between two points falls exponentially with the distance between them. Then the e-folding distance, i.e. the distance where the correlation coefficient has fallen to 1/e = 0.37 for the first time, is conventionally considered to represent the spatial scale of the variable^38^,^39^,^40^,^41^. The current study followed this approach. However, it should be noted that a higher correlation coefficient could also be used to determine spatial coherence^19^.

The exponential function was fitted by the least squares method^42^. In fitting the exponential function we forced a starting point of 1.0, which means that the correlation with the same time series is the highest possible. Further, we excluded all data points after the first negative correlation (the negative correlation associated with the shortest distance) in the fitting process (see Supplementary Fig. S1). We performed the fitting when the number of non-negative correlations is equal or larger than 10. Otherwise, no fitting was not carried out and the scale was defined by the distance between the reference grid box and the first grid box which shows a correlation coefficient lower than the threshold. One such example is demonstrated by Supplementary Fig. S2 which shows that the next grid box from the reference grid box already shows a correlation coefficient that is lower than the threshold, although the correlation went up after that grid box. In that case the scale was approximated by the resolution of the dataset. Since the valid data points were less than ten, no fitting was performed. This procedure resulted in a one-point correlation map in a given direction centered on the reference point. We can repeat the calculation for all possible reference points (all grid boxes in the study area) and any directions of interest. Finally, all the spatial scales estimated can be plotted as a map of the spatial scale in that direction. We choose to focus on the two main directions (W-E and S-N) in this study as shown by Fig. 2.

## Additional Information

**How to cite this article**: Chen, D. *et al*. Satellite measurements reveal strong anisotropy in spatial coherence of climate variations over the Tibet Plateau. *Sci. Rep.* **6**, 30304; doi: 10.1038/srep30304 (2016).

## Supplementary Material

Supplementary Information

## Figures and Tables

**Table 1:** Spatial scales (km) for the annual surface temperature (LST) and precipitation (P) variations in the S-N and W-E directions for the eight ice-core sites.

		LST		P	
		S-N	W-E	S-N	W-E
Typical scale for the TP		302	480	111	182
Ice-cores sites	Muztagata	5	207	8	8
	Guliya	49	196	8	23
	Malan	224	58	8	18
	Puruogangri	301	645	23	26
	Geladaindong	147	234	24	47
	Dunde	36	32	115	69
	Dasuopu	26	78	47	127
	NoijinKangsang	167	371	8	8

The corresponding typical scales for the entire TP are also shown.
