# Supplementary material for: Satellite measurements reveal strong anisotropy in spatial coherence of climate variations over the Tibet Plateau
Source: Sci Rep. 2016 Aug 24;6:30304. doi: 10.1038/srep30304 (PMC4995513; doi:10.1038/srep30304)
Supplement: Supplementary Information [file srep30304-s1.pdf]

Satellite measurements reveal strong anisotropy in spatial coherence of climate variations over the Tibet Plateau

Deliang Chen[a,*], Yudong Tian[b], Tandong Yao[c,*], Tinghai Ou[a]

[a]Regional Climate Group, Department of Earth Sciences, University of Gothenburg, Gothenburg, S-405 30, Sweden

[b]NASA GSFC and ESSIC, University of Maryland, College Park, MD 20742, USA

[c]Institute of Tibetan Plateau Research, Chinese Academy Sciences, Beijing, 100101, China

* Corresponding author**:** tdyao@itpcas.ac.cn or deliang@gvc.gu.se

Table S1. Geographic information about the eight ice-core site.

Site name	Longitude (^{o}E)	Latitude (^{o}N)	Altitude (m)
Muztagata	75.10	38.28	7010
Guliya	81.48	35.28	6200
Malan	90.67	35.83	5680
Puruogangri	89.08	33.92	6070
Geladaindong	91.18	33.34	5720
Dunde	96.40	38.10	5325
Dasuopu	85.72	28.38	7200
Noijin Kangsang	90.20	29.04	5950

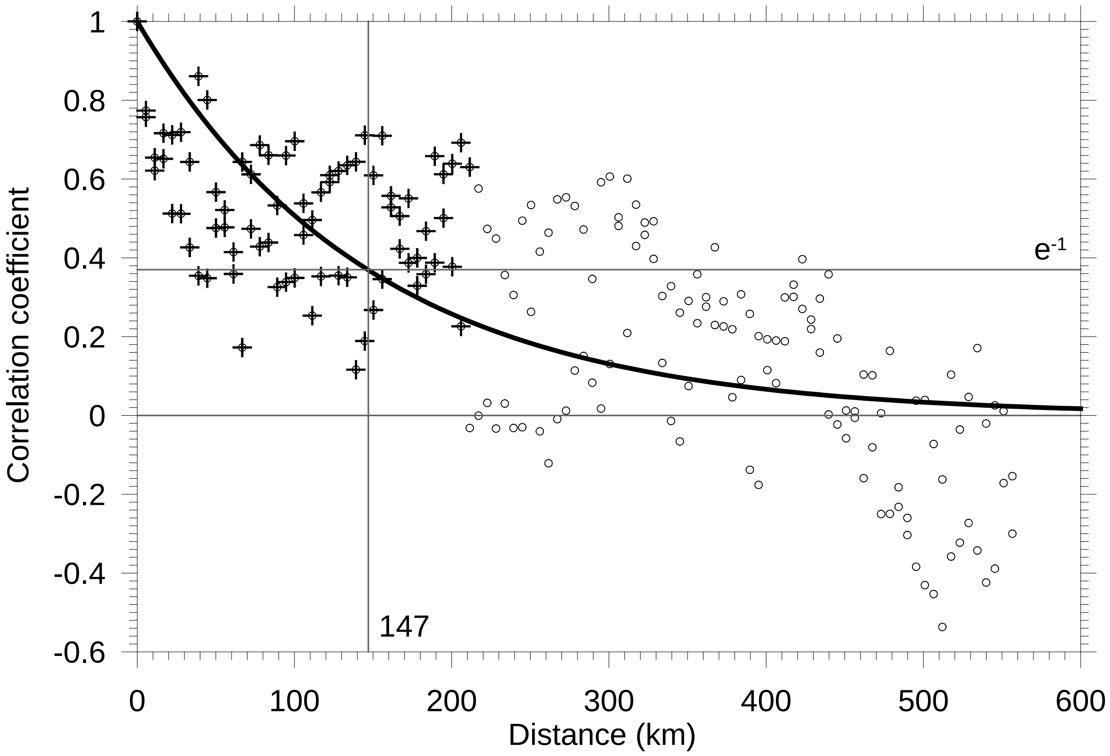

Figure S1. Definition of spatial scale with an example showing the decay of spatial correlation for the annual surface temperature between the reference grid box Geladaindong and all other grid boxes in the south-north direction in the study area as a function of distance. Crosses indicate the points that were used in fitting to the exponential function.

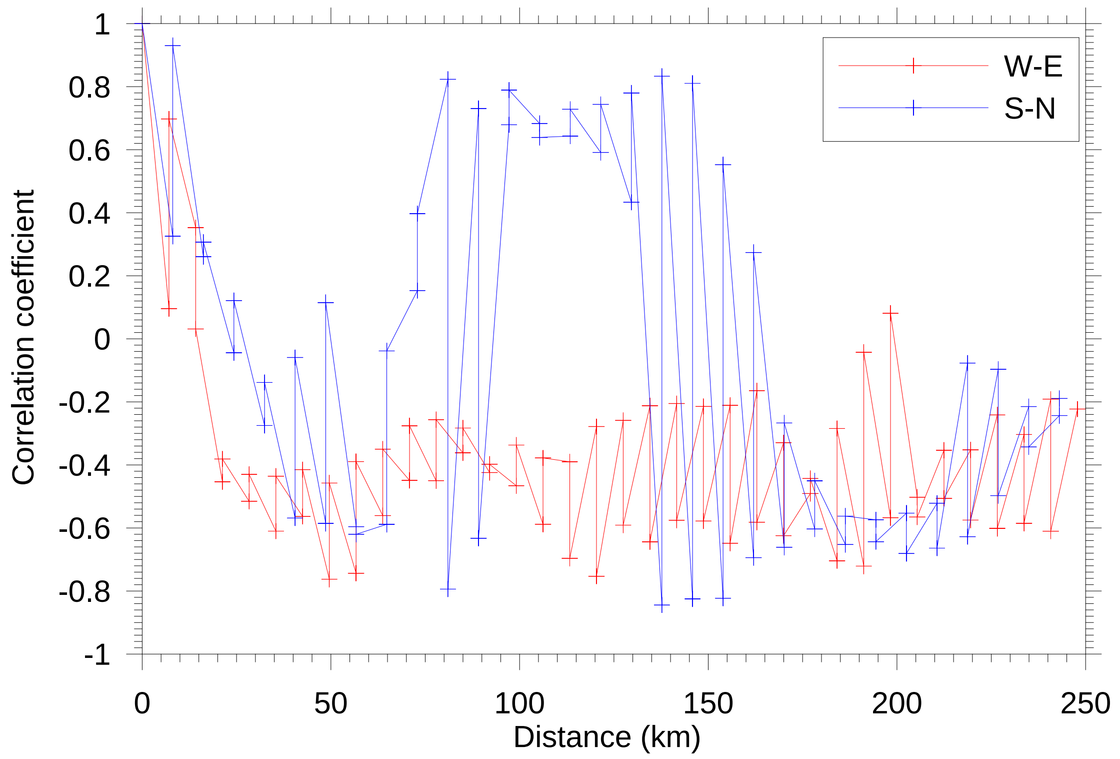

Figure S2. Decay of spatial correlation for the annual precipitation between the reference grid box at Noijin Kangsang and all other grid boxes within 250km along the south-north and west-east direction as a function of distance.

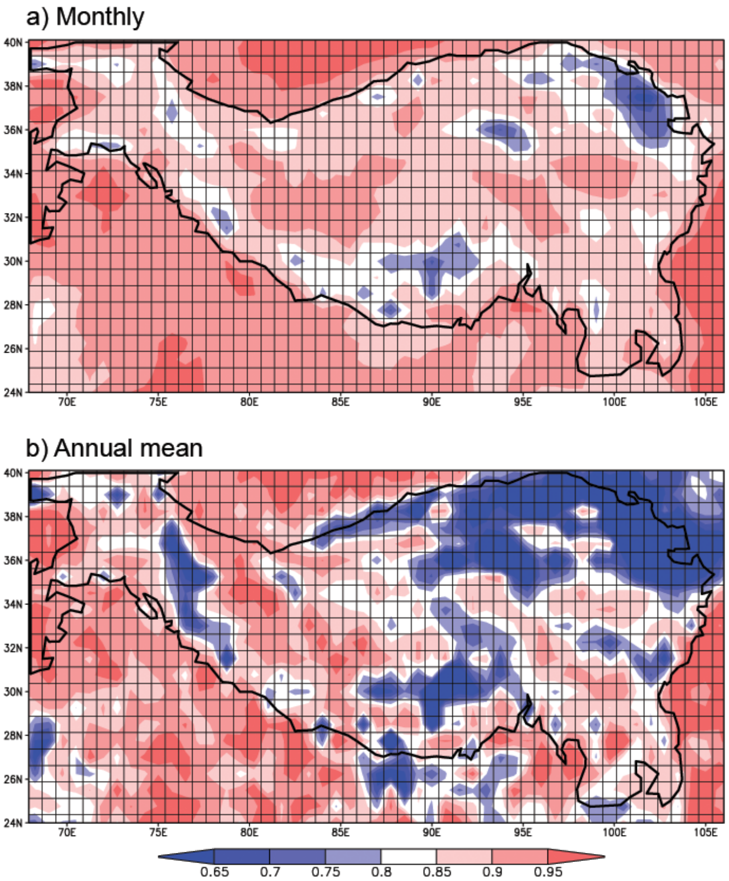

Figure S3. a) Correlation coefficients between the surface temperature and surface air temperature from ERA-Interim during 1979 and 2014. The seasonal cycles of the two monthly time series had been removed before the correlation calculation; b) same as a), but for the annual mean time series. (Figure was created using Grads version 2.0.2, http://iges.org/grads/)
